# Supplementary figures and images for: Structural plasticity of bacterial ESCRT-III protein PspA in higher-order assemblies
Source: Nat Struct Mol Biol. 2024 Aug 16;32(1):23–34. doi: 10.1038/s41594-024-01359-7 (PMC11746142; doi:10.1038/s41594-024-01359-7)

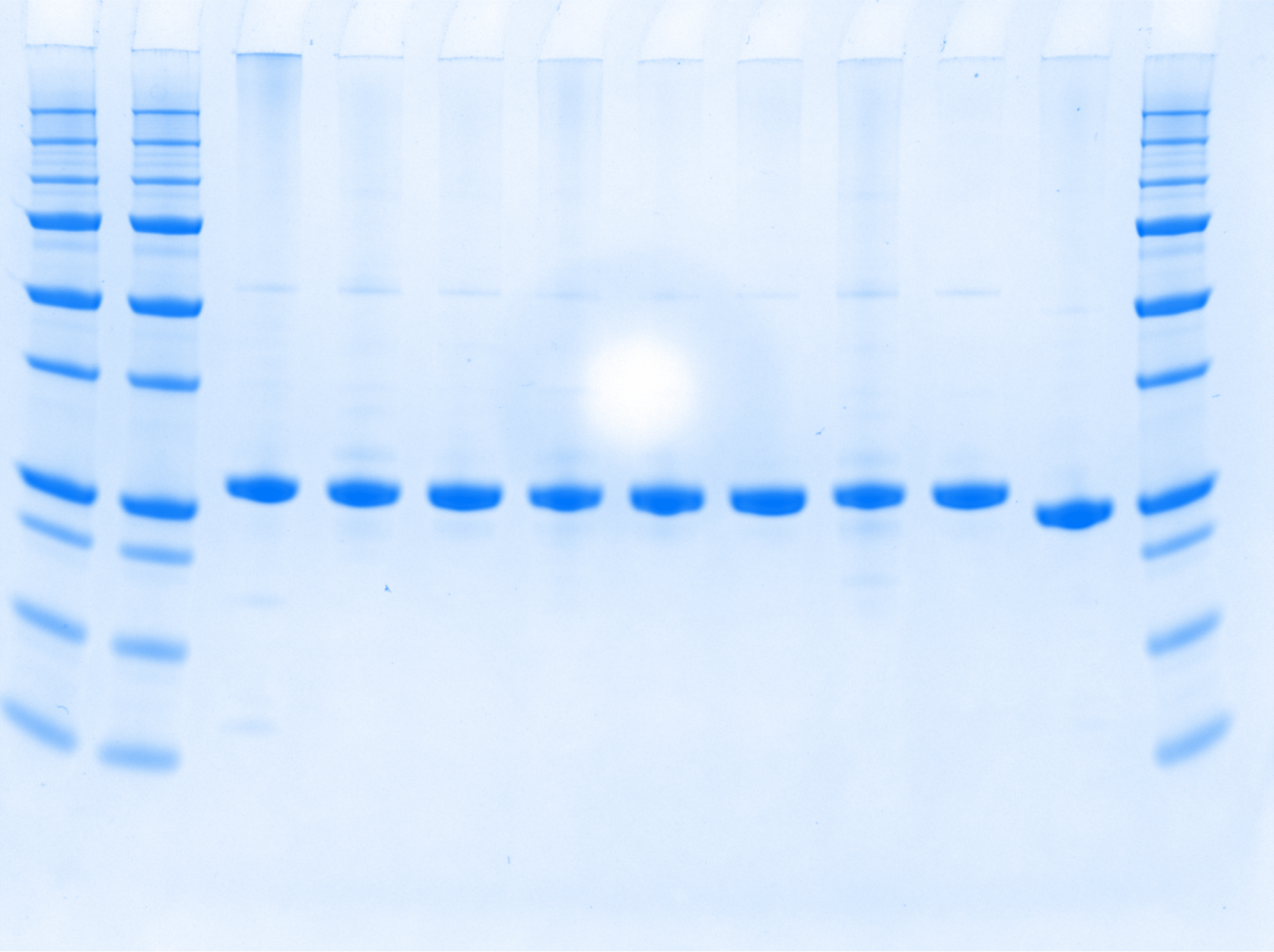

Supplement: Supplementary file 10 — Uncropped gel. [file 41594_2024_1359_MOESM10_ESM.tif]
